# Abdominal obesity and serum adiponectin complexes among population-based elementary school children in Japan: a cross-sectional study

**DOI:** 10.1186/1471-2431-14-81

**Published:** 2014-03-26

**Authors:** Hirotaka Ochiai, Takako Shirasawa, Rimei Nishimura, Hinako Nanri, Tadahiro Ohtsu, Hiromi Hoshino, Naoko Tajima, Akatsuki Kokaze

**Affiliations:** 1 Department of Public Health, Showa University School of Medicine, 1-5-8 Hatanodai, Shinagawa-ku, Tokyo 142-8555, Japan; 2 Division of Diabetes, Metabolism and Endocrinology, Department of Internal Medicine, Jikei University School of Medicine, 3-25-8 Nishi-Shinbashi, Minato-ku, Tokyo 105-8461, Japan; 3 Jikei University School of Medicine, 3-25-8 Nishi-Shinbashi, Minato-ku, Tokyo 105-8461, Japan

**Keywords:** Abdominal obesity, Serum adiponectin complexes, School children, Population-based epidemiological study

## Abstract

**Background:**

There are a limited number of studies regarding the association between abdominal obesity and serum adiponectin complexes (high, medium, and low molecular weight adiponectins) among population-based elementary school children, especially in Japan, where blood collection is not usually performed during annual health examinations of school children. The aim of the present study was to investigate the relationship between abdominal obesity and serum adiponectin complexes among population-based elementary school children in Japan.

**Methods:**

Subjects were all the fourth-grade school children (9 or 10 years of age) in the town of Ina during 2005–2008 (N = 1675). The height, weight, percent body fat, and waist circumference (WC) of each subject were measured. Blood samples were drawn from subjects to measure adiponectin isoform values. Childhood abdominal obesity was defined as “a waist-to-height ratio greater than or equal to 0.5” or “a WC greater than or equal to 75 cm”. The Wilcoxon rank-sum test and the logistic regression model were used to analyze the association between abdominal obesity and each adiponectin isoform value.

**Results:**

Data from 1654 subjects (846 boys and 808 girls) were analyzed. Adiponectin complexes were lower in the abdominal obesity group than in the non-abdominal obesity group regardless of sex. Abdominal obesity significantly increased the odds ratio (OR) for each adiponectin isoform level less than or equal to the median value in boys; the OR (95% confidence interval [CI]) was 2.50 (1.59-3.92) for high molecular weight adiponectin (HMW-adn), 2.47 (1.57-3.88) for medium molecular weight adiponectin (MMW-adn), and 1.75 (1.13-2.70) for low molecular weight adiponectin (LMW-adn). In girls, the OR (95% CI) was 1.95 (1.18-3.21) for HMW-adn, 1.40 (0.86-2.28) for MMW-adn, and 1.06 (0.65-1.70) for LMW-adn.

**Conclusions:**

Abdominal obesity was associated with lower adiponectin complexes and the influence of abdominal obesity varied by adiponectin isoform. Furthermore, the impact of abdominal obesity was larger in boys than in girls. The present study results suggest that prevention of abdominal obesity could contribute to the prevention of lower adiponectin levels, especially in boys.

## Background

Childhood obesity has important consequences on health and well-being both during childhood and in later adult life [1]. For example, cardiovascular risk factors such as hypertension, dyslipidemia, and hyperinsulinemia/insulin resistance, which are known to be associated with obesity in adults, are also associated with obesity in children and adolescents [2]. Moreover, a recent study demonstrated that overweight and obesity in childhood and adolescence have adverse consequences on premature mortality in adulthood [3]. Therefore, childhood obesity is a serious public health problem.

A previous study reported that obese individuals with most of their fat stored in visceral adipose depots generally suffer greater adverse metabolic consequences than those with fat stored predominantly in subcutaneous sites [4]. Visceral adipose tissue (VAT) was shown to be associated with many risk factors for chronic diseases and was shown to be related to glucose metabolism, lipid abnormalities, and hypertension [5]. In fact, epidemiologic studies on the distribution of body fat have shown that greater deposition of central fat is associated with type 2 diabetes, less favorable plasma lipid and lipoprotein concentrations, increased blood pressure, and increased left ventricular mass [6,7]. These studies suggest that the prevention of abdominal obesity (central obesity) is very important.

Central obesity is reported to be associated with adiponectin [8]. Adiponectin is a recently described adipokine that has been recognized to be a key regulator of insulin sensitivity and tissue inflammation [9]. It is specifically and abundantly expressed in adipose tissue [10]. In human plasma, adiponectin circulates in distinct multimeric complexes forming trimeric low molecular weight (LMW), hexameric medium molecular weight (MMW), and oligomeric high molecular weight (HMW) complexes [11]. Several studies have shown the relationship between childhood obesity and adiponectin [12-14]. However, there are a limited number of studies regarding the association between abdominal obesity and each adiponectin isoform (HMW adiponectin [HMW-adn], MMW adiponectin [MMW-adn], or LMW adiponectin [LMW-adn]) among population-based elementary school children, especially in Japan, where blood collection is not usually performed during annual health examinations of school children.

Accordingly, the aim of the present study was to investigate the relationship between abdominal obesity and serum adiponectin complexes among population-based elementary school children in Japan.

## Methods

In addition to the annual national health checkups performed in accordance with the School Health Law of Japan, the town of Ina, located in Saitama Prefecture, Japan, had conducted a unique health-promotion program since 1994. In the program, blood and physical examinations were performed for fourth and seventh graders. The present study was conducted as part of this program.

### Study subjects

Subjects comprised all the fourth-grade school children (9 or 10 years of age) in Ina during 2005–2008. Written informed consent was obtained from each subject’s parent or guardian. This study protocol was approved by the two independent institutional review boards at Showa University School of Medicine and Jikei University School of Medicine.

A total of 1,675 subjects were approached and 13 refused to participate in the program (participation rate: 99.2%). Eight subjects were excluded because of incomplete data. Thus, data from 1,654 subjects (846 boys and 808 girls) were analyzed.

### Anthropometric and biochemical measurements

The height and weight of each subject were measured in the school’s infirmary or in a designated room to protect the subject’s privacy during the procedures. For anthropometric measurements, subjects wore light clothing but no shoes or socks. Height was measured to the nearest 0.1 cm using a stadiometer, and body weight was measured to the nearest 0.1 kg using a scale. Body mass index (BMI) was calculated as weight (kg) divided by height (m) squared. Percent body fat was measured with a bipedal biometrical impedance analysis device (Model TBF-102, Tanita, Tokyo, Japan) to the nearest 0.1%, over light clothing in a standing position. Waist circumference (WC) was measured in a standing position at the navel level while another examiner checked verticality from the side. Waist-to-height ratio (WHtR) was calculated as WC divided by height.

Blood samples were drawn from subjects to measure adiponectin isoform values. Adiponectin isoform values were measured using a commercially available enzyme-linked immunosorbent assay kit (Daiichi Pure Chemical Co. Ltd., Tokyo, Japan) [15].

All measurements were recorded annually from 2005 to 2008.

### Definition of abdominal obesity

Childhood abdominal obesity was defined as a WHtR ≥ 0.5 or a WC ≥ 75 cm according to diagnostic criteria for metabolic syndrome in Japanese children and adolescents [16].

### Data analysis

The Shapiro-Wilk test was used to test the normality of distribution. To compare various characteristics between subgroups (boys vs. girls and non-abdominal obesity group vs. abdominal obesity group), the Wilcoxon rank-sum test was used. Spearman’s correlation coefficients were calculated between anthropometric variables and each adiponectin isoform and among each adiponectin isoform (HMW-adn vs. MMW-adn, HMW-adn vs. LMW-adn, and MMW-adn vs. LMW-adn). The logistic regression model was used to calculate the odds ratio (OR) and 95% confidence intervals (95% CI) of abdominal obesity for each adiponectin isoform (HMW-adn, MMW-adn, or LMW-adn) ≤ the median value. The index “adiponectin levels ≤ median value” was used in a recent study and was shown to be associated with a significantly increased risk of having metabolic syndrome [17]. A *P* value < 0.05 was considered statistically significant. All statistical analyses were performed using Statistical Analysis System software (Version 9.2; SAS Institute Inc., Cary, NC, USA).

## Results

Characteristics were compared between boys and girls. BMI, percent body fat, and WC were significantly higher in boys (median: 16.6 kg/m^2^, 18.4%, and 57.5 cm, respectively) than in girls (16.3 kg/m^2^, 15.8%, and 57.3 cm, respectively). HMW-adn and LMW-adn in girls (median: 2.83 μg/mL and 1.68 μg/mL, respectively) were higher than values in boys (2.65 μg/mL and 1.61 μg/mL, respectively).

Comparisons of characteristics between the non-abdominal obesity group and the abdominal obesity group among boys are shown in Table 1. All anthropometric variables in the abdominal obesity group were significantly higher than in the non-abdominal obesity group. Each adiponectin level was significantly lower in the abdominal obesity group than in the non-abdominal obesity group. WC and WHtR were significantly negatively correlated with each adiponectin isoform value. HMW-adn was significantly correlated with MMW-adn (r = 0.68, *P* < 0.001) and LMW-adn (0.44, *P* < 0.001), while MMW-adn was significantly correlated with LMW-adn (0.33, *P* < 0.001).

**Table 1:** Comparisons of characteristics between the non-abdominal obesity group and the abdominal obesity group (boys)

		**Non-abdominal obesity group (n = 748)**	**Abdominal obesity group (n=98)**	***P*** **value**^**a**^
Age (years)	Median (IQR)	9.0(9.0–10.0)	9.0(9.0–10.0)	0.785
	Mean±SD (95% CI)	9.3 ± 0.5(9.26–9.32)	9.3 ± 0.4(9.2–9.4)	
Height (cm)	Median (IQR)	134.5(130.5–138.7)	137.6(132.8–141.0)	<0.001
	Mean±SD (95% CI)	134.7 ± 5.7(134.3–135.1)	137.3 ± 6.4(136.0–138.6)	
Weight (kg)	Median (IQR)	29.6(26.8–32.8)	41.5(37.7–46.3)	<0.001
	Mean±SD (95% CI)	30.1 ± 4.6(29.8–30.5)	42.3 ± 7.5(40.8–43.8)	
BMI (kg/m^2^)	Median (IQR)	16.4(15.3–17.6)	21.8(20.6–23.8)	<0.001
	Mean±SD (95% CI)	16.5 ± 1.7(16.4–16.6)	22.3 ± 2.7(21.8–22.8)	
PBF (%)	Median (IQR)	17.9(15.3–21.1)	29.5(24.3–32.8)	<0.001
	Mean±SD (95% CI)	18.3 ± 4.2(18.0–18.6)	28.7 ± 5.6(27.6–29.8)	
WC (cm)	Median (IQR)	56.9(54.0–60.1)	73.2(69.5–78.6)	<0.001
	Mean±SD (95% CI)	57.4 ± 4.6(57.0–57.7)	74.7 ± 7.1(73.3–76.2)	
WHtR	Median (IQR)	0.42(0.40–0.44)	0.53(0.51–0.57)	<0.001
	Mean±SD (95% CI)	0.43 ± 0.03(0.42–0.43)	0.54 ± 0.04(0.54–0.55)	
HMW-adn (μg/mL)	Median (IQR)	2.73(1.91–4.00)	2.00(1.40–2.96)	<0.001
	Mean±SD (95% CI)	3.10 ± 1.67(2.98–3.22)	2.34 ± 1.45(2.05–2.63)	
MMW-adn (μg/mL)	Median (IQR)	1.82(1.48–2.19)	1.60(1.36–1.86)	<0.001
	Mean±SD (95% CI)	1.90 ± 0.63(1.86–1.95)	1.63 ± 0.50(1.53–1.73)	
LMW-adn (μg/mL)	Median (IQR)	1.64(1.34–2.03)	1.44(1.18–1.77)	<0.001
	Mean±SD (95% CI)	1.71 ± 0.52(1.67–1.74)	1.55 ± 0.63(1.42–1.67)	

*IQR* interquartile range (25th percentile-75th percentile), *SD* standard deviation, *CI* confidence interval, *BMI* body mass index, *PBF* percent body fat, *WC* waist circumference, *WHtR* waist-to-height ratio, *HMW-adn* high molecular weight adiponectin, *MMW-adn* medium molecular weight adiponectin, *LMW* low molecular weight adiponectin.

^a^Wilcoxon rank-sum test.

Table 2 shows comparison of characteristics between the non-abdominal obesity and the abdominal obesity groups among girls. There were significant differences between groups in all anthropometric variables. Each adiponectin level was lower in the abdominal obesity group than in the non-abdominal obesity group. WC and WHtR were significantly negatively correlated with each adiponectin isoform. HMW-adn was significantly correlated with MMW-adn (r = 0.61, *P* < 0.001) and LMW-adn (0.40, *P* < 0.001), while MMW-adn was significantly correlated with LMW-adn (0.27, *P* < 0.001).

**Table 2:** Comparisons of characteristics between the non-abdominal obesity group and the abdominal obesity group (girls)

		**Non-abdominal obesity group (n = 734)**	**Abdominal obesity group (n=74)**	***P*** **value**^**a**^
Age (years)	Median (IQR)	9.0(9.0–10.0)	9.0(9.0–10.0)	0.775
	Mean±SD (95% CI)	9.3 ± 0.5(9.3–9.4)	9.3 ± 0.5(9.2–9.4)	
Height (cm)	Median (IQR)	134.1(130.0–138.8)	137.2(132.6–143.8)	<0.001
	Mean±SD (95% CI)	134.4 ± 6.2(133.9–134.8)	137.6 ± 7.5(135.9–139.4)	
Weight (kg)	Median (IQR)	28.8(26.0–32.3)	41.5(36.6–47.9)	<0.001
	Mean±SD (95% CI)	29.4 ± 4.7(29.1–29.8)	41.9 ± 7.7(40.1–43.7)	
BMI (kg/m^2^)	Median (IQR)	16.0(15.1–17.3)	21.4(20.7–23.0)	<0.001
	Mean±SD (95% CI)	16.2 ± 1.6(16.1–16.3)	21.9 ± 2.4(21.4–22.5)	
PBF (%)	Median (IQR)	15.4(13.2–18.4)	28.1(26.7–30.9)	<0.001
	Mean±SD (95% CI)	15.8 ± 3.8(15.5–16.0)	29.0 ± 4.8(27.9–30.2)	
WC (cm)	Median (IQR)	56.7(53.9–59.6)	72.1(69.5–77.8)	<0.001
	Mean±SD (95% CI)	57.0 ± 4.5(56.6–57.3)	73.4 ± 6.1(71.9–74.8)	
WHtR	Median (IQR)	0.42(0.40–0.44)	0.53(0.51–0.54)	<0.001
	Mean±SD (95% CI)	0.42 ± 0.03(0.42–0.43)	0.53 ± 0.03(0.53–0.54)	
HMW-adn (μg/mL)	Median (IQR)	2.90(1.89–3.98)	2.08(1.22–3.43)	<0.001
	Mean±SD (95% CI)	3.09 ± 1.59(2.97–3.20)	2.40 ± 1.48(2.05–2.74)	
MMW-adn (μg/mL)	Median (IQR)	1.78(1.45–2.19)	1.60(1.19–2.18)	0.025
	Mean±SD (95% CI)	1.88 ± 0.61(1.83–1.92)	1.72 ± 0.67(1.57–1.88)	
LMW-adn (μg/mL)	Median (IQR)	1.69(1.35–2.07)	1.61(1.24–1.97)	0.195
	Mean±SD (95% CI)	1.75 ± 0.58(1.71–1.79)	1.68 ± 0.59(1.55–1.82)	

*IQR* interquartile range (25th percentile-75th percentile), *SD* standard deviation, *CI* confidence interval, *BMI* body mass index, *PBF* percent body fat, *WC* waist circumference, *WHtR* waist-to-height ratio, *HMW-adn* high molecular weight adiponectin, *MMW-adn* medium molecular weight adiponectin, *LMW* low molecular weight adiponectin.

^a^Wilcoxon rank-sum test.

ORs and 95% CIs of abdominal obesity for each adiponectin isoform level ≤ the median value among boys are shown in Table 3. Significantly increased ORs of abdominal obesity were observed for HMW-adn (OR: 2.50, 95% CI: 1.59-3.92), MMW-adn (2.47, 1.57-3.88), and LMW-adn (1.75, 1.13-2.70). Even when percent body fat was adjusted for in the analysis, abdominal obesity significantly increased the OR for each adiponectin isoform.

**Table 3:** Odds ratios and 95% confidence intervals of abdominal obesity for each adiponectin level ≤ the median value among boys

	**Number (%)**				
**Outcome**	**Non-abdominal obesity group (*n*** **= 748)**	**Abdominal obesity group (*n*** **= 98)**	**OR**	**95% CI**	***P*** **value**
HMW-adiponectin					
≤Median	356(47.6)	68(69.4)	2.50	1.59–3.92	<0.001
>Median	392(52.4)	30(30.6)	1.00		
MMW-adiponectin					
≤Median	358(47.9)	68(69.4)	2.47	1.57–3.88	<0.001
>Median	390(52.1)	30(30.6)	1.00		
LMW-adiponectin					
≤Median	363(48.5)	61(62.2)	1.75	1.13–2.70	0.011
>Median	385(51.5)	37(37.8)	1.00		

*HMW* high molecular weight, *MMW* medium molecular weight, *LMW* low molecular weight, *OR* odds ratio, *CI* confidence interval.

ORs and 95% CIs of abdominal obesity for each adiponectin isoform level ≤ the median value among girls are shown in Table 4. The OR of abdominal obesity was 1.95 (95% CI: 1.18-3.21) for HMW-adn, 1.40 (0.86-2.28) for MMW-adn, and 1.06 (0.65-1.70) for LMW-adn. When percent body fat was adjusted in the analysis, the OR of abdominal obesity was not statistically significant for each adiponectin isoform.

**Table 4:** Odds ratios and 95% confidence intervals of abdominal obesity for each adiponectin level ≤ the median value among girls

	**Number (%)**				
**Outcome**	**Non-abdominal obesity group (*n*** **= 734)**	**Abdominal obesity group (*n*** **= 74)**	**OR**	**95% CI**	***P*** **value**
HMW-adiponectin					
≤Median	357(48.6)	48(64.9)	1.95	1.18–3.21	0.009
>Median	377(51.4)	26(35.1)	1.00		
MMW-adiponectin					
≤Median	365(49.7)	43(58.1)	1.40	0.86–2.28	0.171
>Median	369(50.3)	31(41.9)	1.00		
LMW-adiponectin					
≤Median	367(50.0)	38(51.4)	1.06	0.65–1.70	0.825
>Median	367(50.0)	36(48.7)	1.00		

*HMW* high molecular weight, *MMW* medium molecular weight, *LMW* low molecular weight, *OR* odds ratio, *CI* confidence interval.

In the analysis among all subjects, the sex-adjusted OR and 95% CI of abdominal obesity for each adiponectin isoform level ≤ the median value was calculated. The OR (95% CI) was 2.28 (1.63-3.19) for HMW-adn, 1.94 (1.40-2.70) for MMW-adn, and 1.51 (1.10-2.09) for LMW-adn.

## Discussion

In the present study, the relationship between abdominal obesity and serum adiponectin complexes was investigated among population-based elementary school children in Japan. Adiponectin complexes were lower in the abdominal obesity group than in the non-abdominal obesity group. Furthermore, the OR of abdominal obesity for each adiponectin isoform level ≤ the median value varied with each adiponectin isoform. These results suggest that abdominal obesity was associated with lower adiponectin complexes and that the influence of abdominal obesity on each adiponectin isoform varied. To the best of our knowledge, this is the first study on the association between abdominal obesity and adiponectin complexes among population-based elementary school children in Japan, where blood collection and measurement of WC are not commonly performed in annual health examinations at elementary schools. However, the results of the present study should be discussed carefully.

Some baseline characteristics were significantly different between boys and girls. For example, anthropometric variables were higher in boys than in girls. Furthermore, adiponectin levels were higher in girls than in boys. A recent study reported that sex differences in body composition are present very early in life [18]. In addition, serum adiponectin levels were reported to be higher in girls than in boys [12,17]. Therefore, we analyzed the data separately by sex and then examined the relationship between abdominal obesity and adiponectin complexes.

In this study, adiponectin complexes were lower in the abdominal obesity group than in the non-abdominal obesity group regardless of sex. A previous study showed that adiponectin concentrations diminish as VAT increases in children [14]. In fact, some studies reported that serum adiponectin levels were lower in obese children than in nonobese children [12,13], while levels were reported to be lower in the high-VAT group than in the low-VAT group among adolescents [19]. These results were not inconsistent with our study results.

The OR of abdominal obesity for each adiponectin isoform ≤ the median value varied among HMW-adn, MMW-adn, and LMW-adn in the present study. These results suggest that the impact of abdominal obesity varied on each adiponectin isoform. A previous study reported that the antidiabetogenic and antiatherogenic properties of adiponectin are evident early in life and compromised in youth-onset obesity [19]. Furthermore, among three adiponectin isoforms (HMW-adn, MMW-adn, and LMW-adn), HMW-adn has more biological activity than either MMW-adn or LMW-adn [20,21]. Therefore, abdominal obesity in children leads to lower adiponectin levels, especially HMW-adn levels, which could increase the risk for diabetes and atherosclerosis in the future. In fact, a recent study showed that decreased HMW-adn levels were associated with diabetes [22].

Sex differences regarding the influence of abdominal obesity on adiponectin complexes were observed in the present study; the OR for each adiponectin isoform ≤ the median value among boys was consistently higher than that among girls. These results showed that the impact of abdominal obesity on adiponectin complexes was stronger in boys than in girls. A recent study showed the relationship between obesity and testosterone [23], while another study showed that serum testosterone levels were associated with adiponectin concentrations among children [24]. Furthermore, adiponectin levels were reported to decline with age, in association with changes in sex hormones and growth factors, and this relationship appears to be more pronounced in boys than girls [25]. Therefore, sex differences in the relationship between abdominal obesity and adiponectin levels could be due to sex hormones. Because sex hormones were not measured in the present study, further study will be needed to elucidate sex differences.

The present study has some limitations. First, the blood collection from study subjects was conducted in the morning after eating breakfast, which might affect the data. However, some studies have reported that the level of circulating adiponectin does not change in response to a high-fat meal or 75 g of oral glucose load [26,27]. Accordingly, the present study results may not be affected by postprandial status. The second limitation of our study is the lack of information about pubertal stage (Tanner’s stage), although it might be technically difficult to obtain this information from more than 1,500 population-based children. A previous study showed that the first sign of puberty was testicular growth (≥3 ml) in Japanese boys, attained at a mean age of 10.8 years, and breast development (Tanner stage 2) in girls at a mean age of 10.0 years [28]. Because the mean age of our study participants was 9.3 years, puberty stage was not likely to have a substantial impact on study results. Third, subjects in this study were from one town in Japan. Therefore, it might be difficult to apply the present study results to other populations. Finally, this study used a cross-sectional design. Therefore, the possibility of reverse causality exists.

## Conclusions

The present study showed that abdominal obesity was associated with lower adiponectin complexes, and the influence of abdominal obesity varied with each adiponectin isoform. Furthermore, the impact of abdominal obesity was stronger in boys than in girls. These results suggest that prevention of abdominal obesity could contribute to the prevention of lower adiponectin levels, especially in boys.

## Abbreviations

BMI: Body mass index; CI: Confidence intervals; HMW: High molecular weight; LMW: Low molecular weight; MMW: Medium molecular weight; OR: Odds ratio; VAT: Visceral adipose tissue; WC: Waist circumference; WHtR: Waist-to-height ratio.

## Competing interests

The authors declare that they have no competing interests.

## Authors’ contributions

HO and TS planned this study. RN and HN contributed to improving the study in a meaningful way. HO drafted this manuscript. TS and RN performed the data collection. HH supported the data collection. TS supervised the data collection. HO, TO, and AK contributed to the statistical analysis. NT and AK made substantial contributions to the conception of the present study and revision of the manuscript. All authors read and approved the final manuscript.

## Pre-publication history

The pre-publication history for this paper can be accessed here:

http://www.biomedcentral.com/1471-2431/14/81/prepub
